# Emerging principles of cancer biophysics

**DOI:** 10.12703/r/10-61

**Published:** 2021-07-27

**Authors:** Woong Young So, Kandice Tanner

**Affiliations:** 1 Laboratory of Cell Biology, Center for Cancer Research, National Cancer Institute, National Institutes of Health, Bethesda, MD, USA

**Keywords:** microenvironment, tissue biophysics, rheology

## Abstract

Cancer is a multi-step process where normal cells become transformed, grow, and may disseminate to establish new lesions within the body. In recent years, the physical properties of individual cells and the tissue microenvironment have been shown to be potent determinants of cancer progression. Biophysical tools have long been used to examine cell and tissue mechanics, morphology, and migration. However, exciting developments have linked these physical traits to gene expression changes that drive metastatic seeding, organ selectivity, and tumor growth. Here, we present some vignettes to address recent studies to show progress in harnessing biophysical tools and concepts to gain insights into metastasis.

## Introduction

Cancer arises due to the acquisition of deleterious genetic events and environmental insults^1^. These transformative traits involve growth, migration, aberrant extracellular matrix (ECM) production, and phenotypic plasticity. Moreover, cancer cells can hijack normal stromal cells to aid survival^1,2^. These traits coupled with an acquired ability to evade immune surveillance present a formidable challenge against the goal of complete disease eradication^1^. The “hallmarks of cancer” present these concepts as discretized critical elements required for establishment and progression of disease^1^. However, profound changes in the physical characteristics of tissues such as mechanical properties, cell shape, migration, and topographical cues often accompany these hallmarks. Changes in mechanical properties due to edema and *de novo* synthesis of ECM occur at the primary organ and at distant sites where lesions upset the homeostatic balance within the local microenvironment^1–3^. Stromal remodeling of fibrillar matrix components and neoangiogenesis alter the topographical cues. Finally, at the length scale of a single cell, shape and motility are distinct between normal and tumor phenotypes^1^. The importance of the biophysical cues in normal physiology cannot be understated, as studies have shown that cues such as rheological properties, shear forces due to fluid flow, and surface topography modulate how genes are activated and silenced in key processes involving growth, migration, invasion, and metabolism^4–6^. Preclinical models of pancreatic and breast cancer have shown that the stromal ECM microenvironment influences the therapeutic efficacy of chemotherapy, immunotherapy, and nanoparticle delivery at the primary site^7–9^. In the case of cancer, complementary dissection of the spatiotemporal dynamics of the alterations in biophysical properties along the metastatic cascade is needed to understand differences between indolent and aggressive disease, and also for treatment-responsive and refractory cancers.

## Biophysical cues in tumor metastasis

Cells encounter diverse environmental cues as they transit through the circulatory system before arrest and entry into a distal organ^10–14^. During transit, cells are subjected to forces due to confined geometries and shear forces due to fluid transport of nutrients and exchange of wastes. These external cues can modulate cell shape and may irreversibly deform subcellular organelles such as the nucleus^15–17^. Tumor cells have distinct mechanical properties compared to those of normal cells^15,18^. Moreover, tumor cells are also influenced by the mechanical properties of the tissue at both the primary and the secondary sites^10,19^. A key question is how do tumor cells adapt to the difference in tissue mechanical properties. Tissue mechanics starts with the premise that individual cells and tissues can be mechanically defined by a combination of characteristics of viscous and elastic responses (viscoelasticity) on different length and time scales^20–22^. These physical properties are dynamically linked to alterations in strains, stresses, and osmotic pressures. Tissue geometry and composition are also important factors^6,15,23^. These physical changes have been linked to the modulation of epigenetic and genetic pathways that can influence metastatic potential^17,24^. In addition, tissue mechanics is in part dependent on the protein concentration, crosslinking, and microscale architecture of the ECM microenvironment^2,12,25,26^. Some exciting directions in the field of cancer biophysics have demonstrated that biophysical properties such as cell shape, mechanical phenotype, topography, hemodynamic forces, and pressure are important determinants of cancer progression^12,15,23^. In this commentary, we highlight recent work restricted to the last few years on highlighting the role of physical cues in cancer metastasis employing complex physiologically relevant models that recreate distinct environmental cues.

## Can we use single cell morphology to predict metastasis?

Cell morphology has long been used as an important metric to diagnose cancers^15^. More recently, machine learning and digital pathology have simplified diagnostics where improved sensitivity allows for robust discrimination between subtypes of hematological cancers such as leukemias^27^. With respect to assessing metastasis, recent studies have highlighted that metrics such as cell morphology, mechanical phenotype, and migration might be predictive of invasion and metastatic potential for osteosarcomas and breast, pancreatic, and prostate cancers^28–30^. One study using patient data further illustrated how single cell morphometrics combined with single cell transcriptomics could be predictive of metastasis. Using a combination of *in vivo* models, sophisticated imaging, and hierarchical cluster analysis, single cell morphologies were sufficient to predict the efficacy of tumor engraftment and metastasis in a human xenograft model of breast cancer in mice^31^. Simply, the examination of morphometrics such as nuclear and cell shape of single cell clones of a triple negative breast cancer cell line was sufficient to distinguish which features are predictive of key steps of the metastatic cascade such as intravasation (entry into circulation from primary site), extravasation (exit from circulation to secondary site), and outgrowth in the lung^31^. A correlation between transcriptome and morphology identified 155 genes that were confirmed to be correlated with metastasis and survival in a stratified patient cohort^31^. However, further work will be needed to see if this data can be used to identify a signature that is conserved for other triple negative breast cancer cell lines and patient samples. As this technique can be applied to other tumor models of solid cancers, it provides a platform for further comparative studies. Moreover, it also has the potential to be combined at the time of biopsy where a primary tumor could be processed for single cell analysis. This is exciting, as one critical goal of precision medicine is the ability to predict, at the time of diagnosis, if in a given patient a primary tumor would advance to metastatic disease^32–35^.

## Can we use single cell mechanophenotype as a biomarker of pathology?

Mechanical mapping of isogenic models of cancer progression has revealed that cancer cells are softer when compared to normal cells^18^. As cells need to detach and deform to enter and exit tissues, this softening has been postulated as a key step of metastasis. Thus, one approach is to determine if single cell mechanical phenotype can be predictive of metastatic potential^25,36–38^. One confounding issue is that the cellular mechanical properties depend on the length and temporal scales at which the measurements are performed. They are further dependent on experimental conditions, i.e. if measurements are performed on cells cultured in 2D versus 3D versus suspension, and the choice of matrices when embedded in a hydrogel^18,39^. Using a model of osteosarcoma, Holenstein *et al*. found that relative stiffness of the parental cancer clone compared to that of isogenic weakly and highly metastatic clones was heavily dependent on the technique employed^40^. At the nanometer scale, atomic force microscopy (AFM) measurements determined that the highly metastatic variant was softer when cultured on soft and stiff substrates for one pair of clones but showed the opposite trend for a second clone^40^. Microscale measurements using real-time deformability cytometry revealed that the metastatic clones were softer than the parental cancer clone when examined as a single cell suspension^40^. In these experiments, cellular deformation was used as a metric of stiffness. In a similar vein, recent work exploring a pair of metastatic and non-malignant clones and a pair of dormant and aggressive clones of a breast cancer progression series revealed that the microscale mechanics of both normal and cancer cells are heavily context dependent^39^. Using optical trap-based active microrheology, the authors found that cancer cells were stiffer than the normal counterpart when cultured in hydrogels of ECM similar to *in vivo* breast tissue^39^. However, relative stiffnesses varied when cultured in hydrogels of different types of ECM. These studies are heavily focused on relative stiffnesses, but more recent studies are exploring the full spectrum of rheological parameters where energy dissipation, viscosity, and power law dependence may strengthen the use of mechanical phenotype as a metric that more closely predicts metastatic potential.

## Can we use single cell mechanophenotype to understand drug resistance and organ selectivity?

Within a primary tumor, tumor-initiating cells have also been implicated in drug resistance^41,42^. Intratumoral heterogeneity is also known to be an important factor in metastasis^43^. Moreover, if there is an associated risk of metastasis, advance knowledge of the potential site of the lesion would also be useful in personalized treatment. Using immortalized cell lines, multiple organ-seeking clones have been derived from the same parental cell line where preferential colonization of organs such as bone, brain, and lung is achieved when these clones are injected into mice^34,35,44,45^. These data suggest that individual phenotypes may encode for not only metastasis but also organ selectivity and drug resistance. One way of distinguishing these phenotypes may be defining single cell mechanical properties within a given immortalized cell line or patient-derived cells. Some preliminary data support this premise, as breast cancer cells that preferentially homed to lung and bone showed a differential migration and proliferation when cultured on substrates of different stiffnesses^46^. In addition, single cell mechanical phenotyping identified subpopulations of cells within a treatment-resistant line of patient-derived GBM cells^47^. An active area of research is to assess these phenotypes using multiple platforms such as AFM, Brillouin spectroscopy, and optical and magnetic tweezers in preclinical models^12^. In addition, magnetic resonance elastography and particle tracking microrheology have been recently demonstrated as viable modalities to probe cancer biomechanics in mice^48^. However, it is critical to link these mechanical phenotypes to specific biological pathways. Thus, linking these techniques to other “omic” types of analysis is needed to refine our understanding of cancer mechanobiology as a biomarker for drug resistance and organ selectivity.

One salient factor is that cancer cells exist within an organ ecosystem where tumor cells actively recruit normal stromal cells via paracrine signals to aid tumor growth and dissemination^49,50^. Within this milieu, dynamic and reciprocal interactions between individual cancer and stromal cells collectively drive phenotypic and genotypic changes as a function of tumor progression^50^. In addition, acellular components such as changes in ECM composition, concentration, and architecture are modulated by these *de novo* transformed stromal cells^10,12,49,50^. Infiltrating and resident immune cells and neovascularization are also potent regulators within the milieu^51^. These modifications result in alterations in tissue mechanics, interstitial stresses, and aberrant tissue architecture^52^. Specifically, in many solid tumors, cancer-associated fibroblasts (CAFs) stiffen the ECM microenvironment via secreted factors concomitantly with direct remodeling via contractile forces^49,50^. In breast, lung, and pancreatic cancers, molecular signatures have been identified to aid in the discernment of CAF subtypes^50^. Previous studies have shown that stromal and stem cells show a differential response to tumor-secreted factors^52^. Thus, one idea is that molecular heterogeneities correlate with distinct mechanical phenotypes. Furthermore, a complementary strategy might involve mapping the mechanical properties of the transformed stromal cells in our efforts to understand disease progression.

## Can cell motility be a predictor of metastatic potential in preclinical models?

As tumor cells leave the primary site, one key aspect of metastasis is persistent motility^15^. Multiple studies have looked at differential migration speeds, persistence, and displacement across different cancer lines^15^. The results have been mixed in terms of what is predictive of the establishment of a *de novo* lesion. One reason may be that recapitulating the complex environments of migrating disseminated cancer cells is key to establishing a migratory signature^10^. These environments include conduits such as lymphatic and blood vessels and interstitial tissues^15,23^. These conduits vary in widths, ranging from 10s to 100s of microns and curvatures, where surfaces may be linear, branched, or disordered^23^. Differential shear forces and drag due to fluid flow and glycocalyx on cell surfaces may also be potent factors^23^. Drawing on engineering principles, microfluidics, 3D biomimetic models, and animal models have been used to recreate the complexities, thus providing a platform to evaluate how distinct modes of migration are linked to metastatic potential. In recent work, a migratory signature predictive of metastatic potential was established for immortalized and patient-derived breast cancers using a microfluidics-based platform^53^. Cells were siloed into different categories based on the percentage of a given cell line that was highly migratory in the microfluidic device and a proliferative index based on the percentage of cells that were positive for Ki67 staining. These groups were then assessed for metastatic seeding and outgrowth in the lung, liver, and lymph nodes in mice following subcutaneous and tail vein injections. Complementary transcriptomic analysis revealed that the RAS/MAPK and PI3K pathways were key pathways underpinning this signature. In an effort to understand if therapeutics directed at minimizing migration are effective, the authors also employed microfluidics to screen the efficacy of trametinib (inhibitor of MEK1 and MEK2) and buparlisib (inhibitor of PI3K) in limiting the spread of breast cancer cell lines^53^. They determined that three triple negative breast cancer cell lines were less migratory when treated with trametinib. In contrast, a non-uniform result was obtained following treatment with buparlisib where an increase of migration in one cell line was observed. These data highlighted the fact that one drug does not affect all genotypes similarly for a given phenotype^53^. Moreover, it demonstrated that a “systems approach” in mitigating aberrant signaling might be needed for effective molecular targeting.

One factor that may be missing using *in vitro* biomimetics is the incorporation of physiological shear forces and cell types such as endothelial cells present in many vessel conduits *in vivo*^23^. However, single cell analysis remains technically challenging if visualization and characterization of transit within multiple organs and vascular systems are desired outcomes. Recently, the zebrafish has become useful to evaluate human xenografts of cancer cells *in vivo.* Moreover, patterns of metastasis of human tumors in mouse models are observed in zebrafish^16,17^. The size of zebrafish allows for larger sample sizes which are not readily feasible using mice. The optical properties of the zebrafish also affords the benefit of ease of use of optical-based techniques such as imaging and mechanical mapping. Recently, blood flow was shown to modulate the extravasation potential of human cancer cells in larval fish at the age where the size of blood vessels is comparable to mammalian capillaries^54^. Direct quantitation of the hemodynamic forces and the adhesion force to the luminal surfaces was performed using optical tweezers in this system^54^. Importantly, cell receptors such as β1 integrin were found to be important for “sensing” these external forces during extravasation. In addition to fluid flow, the architecture of the blood vessels was also shown to be an important factor in organ selection^55^. In larval zebrafish, organ selectivity of human breast cancer cell lines is driven by both vessel topography and cell type-dependent extravasation in the larval zebrafish^55^. Specifically, silencing of β1 integrin, a key protein for mechanosensing, reduced extravasation in the bone marrow-seeking clone in the topographically complex vasculature of the zebrafish bone marrow niche. In contrast, silencing the same gene had no effect on the brain-homing clone; instead increased extravasation was observed in the topographically linear brain vasculature. Importantly, these studies belie the importance of external physical cues in regulating organ selection and extravasation, the caveat being that only if the tumor cells have the machinery to interpret these cues can physical properties influence cellular behavior^55^. They also show the importance of multiplexed analysis where biophysical phenotyping is performed in addition to transcriptomic and proteomic analysis. The model system has been used to assess effective therapeutics for patient-derived xenografts^56^. However, elucidation of the role of physical cues in drug efficacy and ultimately a platform for personalized medicine is ongoing.

## Future perspectives: moving to the clinic

In this commentary, we highlight only recent research reports focused on specific biophysical cues in preclinical models. One goal would be to continue to build on findings from basic science to provide a pipeline for clinical use. Many commonly employed diagnostic tools are rooted in our understanding of the interplay between light–tissue interactions and the mechanical properties of tissues^26,38,57^. Imaging modalities such as X-ray, optical coherence tomography, ultrasound, and magnetic resonance imaging are now routine in the clinic^57–59^. These non-invasive techniques detect changes in optical and mechanical properties of tissue associated with disease^57–59^. Specifically, Fibroscan, an ultrasound-based modality, quantitates liver stiffness as a metric to differentiate normal from that of diseased phenotypes such as fibrotic (excess type I collagen deposition and scar formation) and steatosis (due to increase in adipose tissue)^60,61^. Within the framework of cancer, the mammogram, an X-ray-based modality, is routinely used as a screening tool of malignancy where cancer is optically and mechanically distinct from normal tissue^57,59,62–64^. Mammograms are also sensitive to collagen-dense breasts, a feature that is associated with an increased risk of breast cancer^64–67^. These techniques are amenable to multiplexed analysis in combination with other platforms^59,67,68^. Not restricted to diagnosis *in situ*, *ex vivo* analysis of fresh or frozen tissue biopsies using techniques such as atomic force microscopy and optical and magnetic tweezers also supports the notion that tissue mechanics is a reliable hallmark of cancer^38,69–71^. In a similar vein, preclinical analysis of additional biophysical properties can provide a framework that will provide insight into determinants of tumor etiology, cancer progression, and metastasis. These concepts are of critical importance for effective and durable treatment, especially in the event of metastatic disease.
